# Executive control network resting state fMRI functional and effective connectivity and delay discounting in cocaine dependent subjects compared to healthy controls

**DOI:** 10.3389/fpsyt.2023.1117817

**Published:** 2023-02-23

**Authors:** Kyle Woisard, Joel L. Steinberg, Liangsuo Ma, Edward Zuniga, Michael Lennon, F. Gerard Moeller

**Affiliations:** ^1^Institute for Drug and Alcohol Studies, Virginia Commonwealth University, Richmond, VA, United States; ^2^Wright Center for Clinical and Translational Research, Virginia Commonwealth University, Richmond, VA, United States; ^3^Department of Psychiatry, Virginia Commonwealth University, Richmond, VA, United States; ^4^Department of Radiology, Virginia Commonwealth University, Richmond, VA, United States; ^5^Department of Pharmacology and Toxicology, Virginia Commonwealth University, Richmond, VA, United States; ^6^Department of Neurology, Virginia Commonwealth University, Richmond, VA, United States

**Keywords:** cocaine dependence, functional connectivity, effective connectivity, executive control network, delay discounting

## Abstract

Resting state functional magnetic resonance imaging (fMRI) has been used to study functional connectivity of brain networks in addictions. However, most studies to-date have focused on the default mode network (DMN) with fewer studies assessing the executive control network (ECN) and salience network (SN), despite well-documented cognitive executive behavioral deficits in addictions. The present study assessed the functional and effective connectivity of the ECN, DMN, and SN in cocaine dependent subjects (CD) (*n* = 22) compared to healthy control subjects (HC) (*n* = 22) matched on age and education. This study also investigated the relationship between impulsivity measured by delay discounting and functional and effective connectivity of the ECN, DMN, and SN. The Left ECN (LECN), Right ECN (RECN), DMN, and SN functional networks were identified using FSL MELODIC independent component analysis. Functional connectivity differences between CD and HC were assessed using FSL Dual Regression analysis and FSLNets. Effective connectivity differences between CD and HC were measured using the Parametric Empirical Bayes module of Dynamic Causal Modeling. The relationship between delay discounting and functional and effective connectivity were examined using regression analyses. Dynamic causal modeling (DCM) analysis showed strong evidence (posterior probability > 0.95) for CD to have greater effective connectivity than HC in the RECN to LECN pathway when tobacco use was included as a factor in the model. DCM analysis showed strong evidence for a positive association between delay discounting and effective connectivity for the RECN to LECN pathway and for the DMN to DMN self-connection. There was strong evidence for a negative association between delay discounting and effective connectivity for the DMN to RECN pathway and for the SN to DMN pathway. Results also showed strong evidence for a negative association between delay discounting and effective connectivity for the RECN to SN pathway in CD but a positive association in HC. These novel findings provide preliminary support that RECN effective connectivity may differ between CD and HC after controlling for tobacco use. RECN effective connectivity may also relate to tobacco use and impulsivity as measured by delay discounting.

## 1. Introduction

Cocaine dependence is a significant public health concern, with no current Food and Drug Administration-approved therapies available. The study of the brain and behavior of cocaine dependent subjects (CD), and addiction in general, has therefore received a great deal of emphasis in order to inform the development of new treatments (1). One tool available for studying the brain of CD is resting state functional magnetic resonance imaging (fMRI). Resting state fMRI functional connectivity measures the statistical association of the fluctuations in the blood oxygen level dependent (BOLD) signal between distinct brain regions (2, 3), allowing for the analysis of functional brain networks. However, functional connectivity does not inform the directional influence of one region upon another region (i.e., whether region A influences region B, whether B influences A, or both A and B influence each other) (4). Effective connectivity has been defined as the strength of the directional coupling between brain regions (4). Resting state fMRI-based effective connectivity can be measured by dynamic causal modeling (DCM) which models at the neural level the excitatory or inhibitory directional connectivity that generates the observed functional connectivity among brain regions or networks (4–7).

Three brain networks which have frequently been proposed to relate to addiction and other psychopathologies are the default mode network (DMN), salience network (SN), and executive control network (ECN – lateralized as left (LECN) and right (RECN) components) (8, 9). Functional connectivity within and among the DMN, SN, and ECN may be altered in substance use disorders, possibly relating to increased incentive salience for interoceptive signals of craving at the expense of executive control (9–12). However, most resting state fMRI functional connectivity studies of CD (and addictions in general) to-date have focused on the DMN (12) with fewer studies assessing functional connectivity of the ECN, despite well-documented executive control behavioral deficits in addictions (13). Recently, we investigated the DMN, SN, and ECN in opioid use disorder subjects and found that these subjects had weaker functional connectivity within the LECN relative to non-drug using control subjects (14). Two other studies found weaker functional connectivity within the LECN in cocaine use disorder (15) and alcohol use disorder (16) relative to control subjects. In contrast, Zhu et al. (17) found stronger functional connectivity within the LECN in alcohol use disorder subjects relative to control subjects. A possible reason for the discrepant findings is that the region showing stronger LECN functional connectivity reported in Zhu et al. (17) was in the posterior parietal cortex, while the region with weaker LECN functional connectivity reported in Woisard et al. (14) was in the left dorsolateral prefrontal cortex. Weiland et al. (16) and Reese et al. (15) did not report which sub-region within the LECN had weaker functional connectivity in patients relative to controls. Reese et al. (15) found weaker functional connectivity between the LECN and SN but stronger functional connectivity between the RECN and SN in cocaine use disorder subjects relative to controls. In contrast, Zhu et al. (17) found stronger functional connectivity between the LECN and SN in alcohol use disorder subjects relative to controls. Schmaal et al. (18) found that alcohol dependent subjects had weaker functional connectivity between the LECN and SN, between the RECN and SN, and between the DMN and SN after taking modafinil relative to placebo, which was not observed for control subjects, but there were no significant differences in functional connectivity between patients and controls prior to treatment.

Resting state fMRI-based effective connectivity among the ECN, SN, and DMN in substance use disorders has not been frequently studied. Our group found that cannabis users had stronger effective connectivity from a medial prefrontal cortex node of the DMN to a right anterior insula node of the SN, although this finding was not replicated in a second dataset (19). Additionally, Goulden et al. (20) showed that the SN modulated effective connectivity between the DMN and ECN in healthy control subjects, consistent with the hypothesized role of the SN in driving the switching between the DMN and ECN; however, their results have yet to be replicated in substance use disorder subjects.

It is also unclear how ECN functional connectivity relates to executive cognitive functions in substance use disorder subjects. In assessing the spatial and functional correspondence between functional brain networks at rest and during different behavioral tasks from the Brainmap database (21), Smith et al. (22) found lateral frontoparietal networks comprising the LECN and RECN to correspond to task-based fMRI regions of activation during multiple executive function tasks. The LECN functional connectivity showed higher relative spatial correspondence with activation from cognitive language tasks and working and explicit memory tasks. The RECN showed higher relative spatial correspondence with activation from action inhibition and working memory tasks. Both networks showed more moderate relative spatial correspondence with activation from a cognitive reasoning task. However, the correspondence of tasks listed in the Smith et al. (22) paper represented a relatively small subset (20 tasks) that showed the strongest associations between resting state functional connectivity and task activation among the 66 behavioral tasks studied, implying that the majority of tasks did not show as good of spatial correspondence with the resting state functional networks. Laird et al. (23) used independent component analysis to find intrinsic connectivity networks from activation studies from the Brainmap dataset (21). They found an intrinsic connectivity map composed of frontoparietal regions (spatially comprising the LECN) to be associated with tasks in the domains of language, working and explicit memory, and reasoning (21). A functional connectivity network comprising the RECN had more moderate task associations which included working memory, action-inhibition, and delay discounting tasks (23). In an aim to replicate the Smith et al. (22) findings, Nickerson (24) assessed the correspondence between resting state and the activation strength of task-based networks from the Human Connectome Project (25) during behavioral tasks from several domains. Nickerson (24) found that the LECN showed relatively greater activation during a relational matching task, working memory task, and a language task (the contrast of a math condition minus a story condition), while the RECN showed greater activation during the working memory task and the language task (24).

The present study used FMRIB Software Library (FSL)^1^ MELODIC ICA to identify the DMN, SN, LECN, and RECN in CD and healthy non-drug using control subjects (HC). ICA allows for model-free estimation of the spatial extent of functional brain networks (2). The degree of functional connectivity in the ICA-estimated brain networks can be further analyzed using the dual regression procedure in the FSL software which considers both the amplitude and shape of the signal time-course and can better account for head motion artifacts than seed-based methods (2, 26). FSL dual regression was used to evaluate the within-network functional connectivity differences between CD and HC. The FSLNets software^2^ was used to evaluate the between-network functional connectivity differences between CD and HC.

The present study also used the Parametric Empirical Bayes (PEB) module of DCM (27) to estimate effective connectivity differences between CD and HC among the DMN, SN, LECN, and RECN. The present study also used DCM to test whether the SN modulated the effective connectivity between the DMN and LECN and between the DMN and RECN. The present study also tested for associations of connectivity with delay discounting task scores across all subjects. Delay discounting is a measure of impulsivity and reflects the tendency to discount the value of a reward if it is delayed in time (28). Higher rates of delay discounting reflect higher impulsivity (28, 29). Delay discounting has been linked to executive control and has been associated with different addictions (30, 31), but has not been well-studied in association with functional and effective connectivity in CD.

Based on the previous literature outlined above, all of the following hypotheses regarding functional and effective connectivity were registered with the Open Science Framework (OSF) [(32), March 7; (33), August 11] prior to any data analysis in this study. Regarding functional connectivity, we hypothesized that CD would have weaker within-network LECN functional connectivity relative to HC. We also hypothesized that CD subjects would have stronger within-network DMN functional connectivity relative to HC. We hypothesized that delay discounting scores would negatively correlate with within-network LECN functional connectivity. We also hypothesized that CD subjects would have stronger functional connectivity between the LECN and SN, weaker functional connectivity between the RECN and SN, and stronger functional connectivity between the DMN and SN relative to HC. We also examined the within-network functional connectivity of the RECN and SN and examined the functional connectivity between the ECN and DMN and between the LECN and RECN in exploratory analyses. We also examined whether impulsivity measured by a delay discounting task correlates with functional connectivity within the RECN, DMN, and SN and functional connectivity between the SN and ECN, SN and DMN, ECN and DMN, and LECN and RECN in exploratory analyses.

Regarding effective connectivity, we hypothesized that the dynamic causal model of the SN modulating the effective connectivity between the DMN and ECN would be the optimum model among those we tested in HC, as found in the Goulden et al. (20) study in healthy control subjects, and that this finding would be replicated in CD. We also hypothesized that the effective connectivity between the LECN/RECN and DMN, LECN/RECN and SN, and SN and DMN would be different in CD compared to HC. We also examined self-connectivity differences between groups for each network in exploratory analyses (34). In addition to the registered hypotheses, we also included whether the CD subject’s urine drug screen on the day of their MRI scan was positive for cocaine or cannabis as factors in a general linear model, in order to address the effects of recent cocaine and cannabis use within the CD group.

## 2. Materials and methods

### 2.1. Subjects and procedures

All study procedures were approved by the Institutional Review Board of Virginia Commonwealth University. CD and HC were recruited from Richmond, Virginia, via flyers, advertisements, and in-person recruitment at outpatient addiction treatment clinics (CD only). CD were excluded if they tested positive for any illicit drug other than cocaine or cannabis, but no restrictions regarding cocaine or cannabis use were imposed during recruitment. Written informed consent was obtained from all subjects. Subjects underwent screenings for medical, psychiatric, and substance use histories, and a physical examination. The Structured Clinical Interview for DSM-IV [(35); SCID-IV] was used to diagnose DSM-IV Cocaine Dependence (36). Inclusion criteria were DSM-IV diagnosed Cocaine Dependence (for CD) and age between 18 and 70 years. Exclusion criteria were history of schizophrenia, seizure disorder, major head trauma, any changes to psychoactive medications within the previous 30 days, or any other DSM-IV substance use disorder diagnosis. Additional HC exclusion criteria were any history of substance use disorder. Subject data was pooled from three separate studies – two studies in which delay discounting and MRI measures were obtained during a baseline period and one study in which the delay discounting and MRI measures were obtained two hours after administration of a placebo dose in a mirtazapine medication study (i.e., subjects had received either no mirtazapine dose or a single low mirtazapine dose 7 days prior to the measurement of delay discounting and MRI data used for this study). Participants were asked to refrain from tobacco use one hour before and caffeine consumption 3 hours before their MRI scan. Urine drug screens (UDS) and alcohol breath screens were obtained before their MRI scan on the day of the scan. 28 CD and 28 HC met the inclusion and exclusion criteria. Given that these two groups differed statistically in mean age and also in mean years of education attained, we performed a planned analysis after matching the two groups more closely for age and years of education. This more closely matched group analysis included 22 CD and 22 HC. We included an equal number of subjects in each group per the recommendations of the authors of the FSL software which we used for our functional connectivity analysis [(37), p. 67].

### 2.2. Behavioral measures

Delay Discounting Task: A 5-trial adjusted delay discounting task (28) was used to measure delay discounting. A subject’s temporal discounting rate is calculated as a “k” value (29). A higher temporal discounting rate (i.e., a higher “k” value) is associated with greater impulsivity (29). The logarithm of the k value [log_10_(k)] is calculated to obtain a more normal distribution across subjects (29).

Cocaine and Cannabis Use: The number of subjects with UDS positive for cocaine and cannabis are reported for descriptive purposes.

Tobacco use was assessed by the Fagerström Test of Nicotine Dependence (38). Subjects were classified as current tobacco users if they responded that they had used tobacco products within the past year, or non-current tobacco users if they responded that they had not used tobacco products within the past year.

Behavioral data were analyzed using the JMP statistical software package (JMP, Version 14. SAS Institute Inc., Cary, NC, 1989-2019). A two-sample *T*-test was performed to test for statistical significance between groups with respect to age, education, tobacco use, head motion (mFD score), and delay discounting task scores.

### 2.3. MRI acquisition

MRI scans were acquired using the Philips Medical Systems (Best, Netherlands) Ingenia wide-bore dStream 3.0 T MRI scanner, with a 32-channel receive head coil. Single shot gradient-echo echoplanar imaging (EPI) was used for acquiring fMRI data. The fMRI acquisition parameters were: repetition time 1500 ms, echo time 30 ms, flip angle 68°, field of view 240 mm (anterior-to-posterior) × 240 mm (left-to-right) × 143.67 mm (foot-to-head), in-plane resolution 3.75 mm × 3.75 mm, 32 axial slices, slice thickness 3.75 mm, interslice gap 0.76 mm, 375 repetitions per run after 12 dummy acquisitions, and total duration 9 minutes. During the resting state fMRI scan, subjects were asked to look at a black fixation cross on a white screen. A T1-weighted 3-Dimensional Magnetization Prepared Rapid Gradient Echo (3D-MPRAGE) scan with acquisition voxel size = [1 × 1 × 1] mm and 160 sagittal slices was acquired for offline co-registration with the fMRI scans, and a T2-weighted Fluid-Attenuated Inversion Recovery (FLAIR) scan was read by a neuroradiologist to screen for incidental pathology.

### 2.4. MRI preprocessing

Preprocessing was conducted similarly to Woisard et al. (14). Initial removal of signal outliers, spatial smoothing, registration to a T1-weighted anatomical scan, and slice timing correction were performed. Quality control for head motion was conducted by excluding subjects whose fMRI runs did not meet the Parkes et al. (39) stringent criteria (if the mean relative framewise displacement (mFD) was greater than 0.20 mm, if the number of timepoints with framewise displacements (FDs) greater than 0.25 mm was greater than 20% of the total number of volumes in the run, if any individual FD was greater than 5 mm, or if the run contained less than 4 continuous minutes without any FDs above 0.25 mm). Head motion re-alignment was performed using FSL MCFLIRT (40, 41). Head motion signal correction was conducted with ICA-AROMA (42). Subsequent further signal denoising was performed using aCompCor for CSF and white matter implemented in the CONN software [(43), RRID:SCR_009550].^3^ High pass filtering (cutoff period = 125 s) was performed as a final step after all other denoising steps had been completed. Furthermore, group ICA signal components with possible motion-related or other structured noise were regressed out as part of the FSL dual regression step [(37), pp. 64–65]. The T1-weighted anatomical scan and the denoised fMRI timeseries were transformed into MNI space using the FSL non-linear transformation module FNIRT.

### 2.5. Functional connectivity analysis

The within-network functional connectivity analysis was conducted as described in Woisard et al. (14). Group ICA was conducted with FSL Multivariate Exploratory Linear Optimized Decomposition into Independent Components [MELODIC; (44)]^4^ with a dimensionality of 30 (i.e., 30 independent components), in order to match the dimensionality of our previously conducted study in opioid use disorder (14). The output was inspected visually to identify the DMN, SN, LECN, and RECN, based on previous published studies (8, 14, 45, 46). The dual regression procedure (26) implemented in FSL was then used to generate a subject-specific timecourse for each of the DMN, SN, LECN, and RECN network components, and a subject-specific spatial map for each network component containing the functional connectivity strength at each voxel. These subject-specific spatial maps were compared between groups to assess differences in within-network functional connectivity. The FSL standard Threshold Free Cluster Enhancement (TFCE) was used to assess for statistically significant clusters of voxels while keeping family-wise-error (FWE) control for multiple comparisons (47), using the FSL default setting with parameter values of *H* = 2 and *E* = 0.5. The subject-specific timecourses were input into the FSLnets program (see text footnote 2) to estimate between-network functional connectivity between each network pair using partial correlation coefficients. The use of partial correlation coefficients allows for more direct estimation of connectivity between network pairs by regressing out the timeseries of all other networks. These resulting partial correlation coefficients were Fisher’s r-to-Z transformed, and the subject-specific Z-statistic for each network pair (referred to as an “edge”) was compared between groups to assess between-network functional connectivity. Functional connectivity group differences and regression analyses were conducted using FSL’s Permutation Analysis of Linear Models (PALM) (48).^5^ Rigorous FWE correction for multiple comparisons across voxels in the brain, the two contrasts examined for each test, and the number of networks or network pairs examined for each test was conducted using the PALM program for the functional connectivity analysis. The anatomical locations of statistically significant voxels were determined using the Harvard-Oxford Cortical Structural Atlas.^6^

### 2.6. DCM analyses

#### 2.6.1. Stochastic DCM of non-linear modulatory effects of each network on the effective connectivities between the other networks

Following Goulden et al. (20), the subject-specific timeseries that were output from Dual Regression stage 1 for the SN, DMN, LECN, and RECN were used as the input for the DCM effective connectivity analysis. The Stochastic module of DCM (49) was conducted, which improves, relative to deterministic DCM, the calculation of the non-linear modulatory effects of a given node on the effective connectivities between the other nodes. We first assessed whether the Goulden et al. (20) findings of the SN modulating the effective connectivity between the DMN and ECN could be replicated in HC and whether those findings differed between HC and CD. We accomplished this by estimating and comparing four different non-linear models for each subject: each model differed in terms of which network was posited as modulating the connections between the other networks. For example, in model #1, the SN putatively modulated the connections between the DMN and LECN, and between the DMN and RECN. In each subsequent model, one of the other networks in turn is posited as being the modulator of the connections between the other networks. The optimum model among those tested was determined by Bayesian Model Selection (50), following Goulden et al. (20). Protected exceedance probabilities and Bayesian Omnibus Risk (BOR), which quantifies the probability that the model frequencies are equal, are reported (51).

#### 2.6.2. Spectral DCM without non-linear modulation of a network on the effective connectivities between the other networks

If the results of stochastic non-linear DCM analysis did not show superiority for any one network as modulating the connections between the other networks, we proceeded with Spectral DCM (7), which may provide more accurate results than Stochastic DCM for resting state fMRI in the absence of non-linear modulation in the model (7). We compared the effective connectivities across subjects using the DCM second-level Parametric Empirical Bayes (PEB) module (27). The Bayesian posterior probabilities (Pp) and the effective connectivity strengths in Hertz (Hz), which were calculated by the PEB procedure, are reported for the mean connectivity across all subjects and for group differences in connectivity. A group effective connectivity difference or effective connectivity regression was considered strong evidence if the Bayesian Pp was greater than or equal to 0.95, following Ma et al. (19).

### 2.7. Analyses of relationships between behavioral measures and functional and effective connectivity in CD and HC

The association between delay discounting and functional and effective connectivity was assessed by regression of delay discounting task scores [log_10_(k)] on functional and effective connectivity.

For baseline categorical variables for which there was a significant difference between groups (the baseline current tobacco use status), we included current tobacco use status in a multifactorial general linear model (GLM) analysis of variance (ANOVA). Thus, the model consisted of the factors: group (two levels: CD and HC), current tobacco use status (two levels: current tobacco user and non-current tobacco user), and the interaction of group x current tobacco use status.

To examine the effects of heterogeneity in our CD sample, we also compared CD with cocaine-positive UDS to CD with cocaine-negative UDS. We also compared CD with cannabis-positive UDS to CD with cannabis-negative UDS.

We also conducted a regression analysis of functional connectivity on head motion (mean framewise displacement – mFD) as a quality control measure to assess whether head motion was statistically significantly related to functional connectivity.

## 3. Results

### 3.1. Demographic and behavioral results

#### 3.1.1. Demographics

The mean, standard deviation, and range of age, years of education attained, and mFD of CD and HC are listed in Table 1. Age did not significantly differ between groups, (*t* = 1.53, df = 42, *p* = 0.134). Years of education did not significantly differ between groups (*t* = 1.62, df = 42, *p* = 0.112). mFD did not significantly differ between groups (*t* = 1.04, df = 42, *p* = 0.302). 5 out of the 22 CD were female and 7 out of the 22 HC were female. A Chi-Square Test determined the groups did not differ significantly in sex (*X*^2^ = 0.46, df = 1, *p* = 0.498). 17 out of 20 CD self-reported smoking tobacco products within the last 12 months and were classified as current tobacco users compared to 7 of the 22 HC (*t* = 4.02, df = 40, *p* = 0.0002). Two CD subjects did not have tobacco use data recorded.

**TABLE 1:** Demographic information.

	HC (*n* = 22)		CD (*n* = 22)		Difference
	**Mean (SD)**	**Range**	**Mean (SD)**	**Range**	**Statistic**
Age	39.8 (11.0)	24 to 57	44.6 (9.9)	27 to 59	*t* = 1.53, df = 42, *p* = 0.134
Education	13.8 (2.0)	11 to 17	13.0 (1.7)	11 to 18	*t* = 1.62, df = 42, *p* = 0.112
mFD	0.08 (0.04)	0.03 to 0.20	0.096 (0.05)	0.05 to 0.20	*t* = 1.04, df = 42, *p* = 0.303
DDT log_10_(k)	−2.54 (1.10)	−4.55 to −0.58	−1.57 (1.08)	−4.06 to 1.21	*t* = 2.82, df = 38, *p* = 0.0075**
Ethnicity	15 AA, 5 C, 1 H, & 1 mixed		22 AA		
Sex	7 F		5 F		*X*^2^ = 0.46, df = 1, *p* = 0.498
Current tob. use status	7/22 users		17/20 users		*t* = 4.02, df = 40, *p* = 0.0002**

DDT, delay discounting task; X^2^, Chi-Square statistic; mFD, mean framewise displacement; C, Caucasian; H, Hispanic; AA, African American; A, Asian; F, female, Tob., tobacco. **p < 0.01. Two CD subjects did not have tobacco use status data recorded.

#### 3.1.2. CD urine drug screens

13/22 CD subjects tested positive for cocaine. 9/22 CD subjects tested positive for cannabis. 7/22 CD subjects tested positive for both cocaine and cannabis. 7/22 CD subjects tested negative for both cocaine and cannabis. UDS results for CD subjects are listed in Table 2.

**TABLE 2:** Urine drug screen (UDS) positive results in cocaine dependent subjects (CD).

	Number of subjects with positive UDS
Cocaine	13 out of 22
Cannabis	9 out of 22
Cocaine and cannabis	7 out of 22
Negative	7 out of 22

UDS, urine drug screen.

#### 3.1.3. Behavioral results

The mean, standard deviation, and range of Delay Discounting log_10_(k) scores of CD and HC subjects are listed in Table 1. Two subjects in each group (four subjects total) did not have delay discounting log_10_(k) scores recorded. CD subjects scored significantly higher in Delay Discounting log_10_(k) scores than HC subjects (*t* = 2.82, df = 38, *p* = 0.0075).

### 3.2. Functional connectivity results

#### 3.2.1. Within-network functional connectivity between groups

**FIGURE 1:**
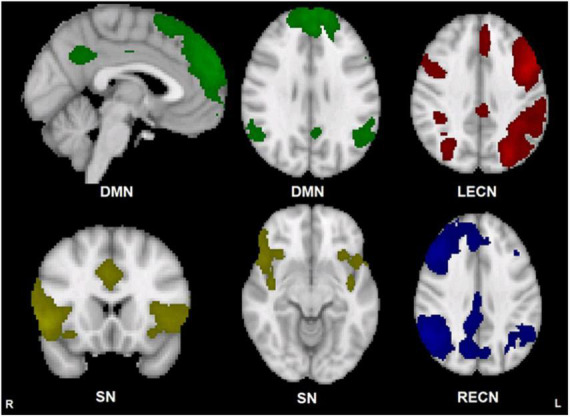
Group template maps generated from FSL MELODIC ICA for all subjects thresholded arbitrarily at *Z* ≥ 3 for display purposes. Units are Z-scores calculated by dividing the original component connectivity strength at each voxel by the standard deviation of the residual noise. The left side of the brain is on the viewer’s right side for the axial and coronal images. Color depictions and MNI coordinates (mm) of the slice location of each image: DMN – green [sagittal slice: *x* = –2], [transverse slice: *z* = 29], SN – yellow [coronal slice: *y* = 18], [transverse slice: *z* = –10], LECN – red [transverse slice: *z* = 36], RECN – blue [transverse slice: *z* = 36].

Component maps for the DMN, SN, LECN, and RECN, generated by FSL MELODIC from both groups combined, are displayed in Figure 1. There were no significant group differences in within-network functional connectivity for any of the 4 networks examined (p greater than 0.071).

#### 3.2.2. Between-network functional connectivity between groups

CD and HC did not significantly differ in the between-network functional connectivity for any of the 6 possible network pairs (FWE p greater than 0.674).

#### 3.2.3. Delay discounting regression analyses

The regression of Delay Discounting log_10_(k) values on within-network functional connectivity was not significant for any of the 4 networks or 6 possible between-network pairings (FWE p greater than 0.166) nor were there any significant log_10_(k) x group interaction effects in any of the 4 networks or 6 possible between-network pairings (FWE p greater than 0.929).

#### 3.2.4. Head motion regression analyses

The negative regression of mFD on SN within-network functional connectivity was statistically significant (FWE p less than 0.05) for three clusters located in the right hemisphere of the brain (peak voxel FWE p = 0.022). The positive regression of mFD on SN within-network functional connectivity was not significant. The positive or negative regression of mFD on within-network functional connectivity was not significant for any of the other 3 networks, or 6 possible between-network pairings (FWE p greater than 0.180). There were no significant mFD x group interaction effects (FWE p greater than 0.723).

#### 3.2.5. UDS regression analyses

CD with cocaine-positive UDS did not differ from CD subjects with cocaine-negative UDS in functional connectivity within any of the 4 networks or between any of the 6 network pairs examined (FWE p greater than 0.280). CD subjects with cannabis-positive UDS did not differ from CD subjects with cannabis-negative UDS in functional connectivity within any of the 4 networks or between any of the 6 network pairs examined (FWE p greater than 0.365).

#### 3.2.6. Tobacco use multifactorial ANOVA

There were no significant current tobacco use x group interaction effects in any of the 4 networks or the 6 possible between-network pairings (FWE p greater than 0.300). The main effects of current tobacco use status on within-network functional connectivity were not significant for any of the four networks or six possible between-network pairs (FWE p greater than 0.059). The main effects of group in the Multifactorial ANOVA were not significant for any of the 4 networks or 6 between-network pairs (FWE p greater than 0.318).

### 3.3. DCM results

#### 3.3.1. Stochastic DCM of non-linear modulatory effects of each network on the effective connectivities between the other networks

The Bayesian Model Selection results for HC subjects are displayed in Supplementary Table 1. None of the 5 models had a protected exceedance probability more than marginally greater than the others, and the probability of equal model frequencies was BOR = 0.98. Results for CD subjects were similar (BOR = 0.97) and are also displayed in Supplementary Table 1.

#### 3.3.2. Spectral DCM without non-linear modulation of a network on the effective connectivities between the other networks

##### 3.3.2.1. Spectral DCM PEB results – Mean connectivity across all subjects

The PEB second-level GLM results across all subjects are reported in Supplementary Table 2, and the analysis showed strong evidence for negative effective connectivity different from zero for the SN to DMN pathway, for the DMN to RECN pathway, for the LECN to RECN pathway, for the SN to SN self-connection, for the DMN to DMN self-connection, and for the RECN to RECN self-connection.

##### 3.3.2.2. Spectral DCM PEB results – Group differences

The PEB second-level GLM results are reported in Supplementary Table 2, and the analysis did not show strong evidence for group differences in effective connectivity strength between CD and HC.

##### 3.3.2.3. DCM PEB results – Delay discounting

**FIGURE 2:**
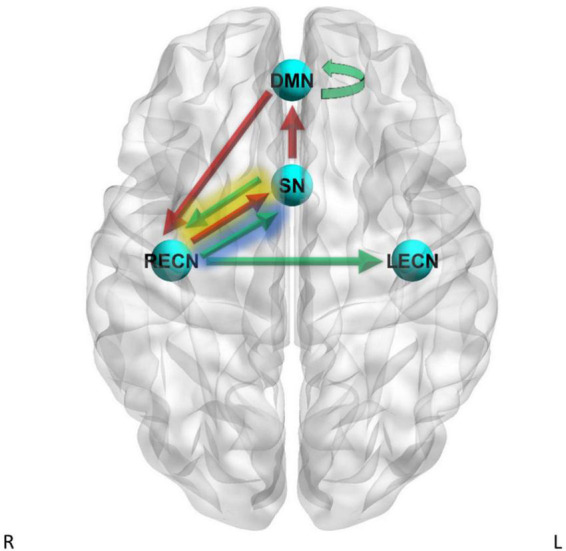
Dynamic causal modeling (DCM) PEB results – delay discounting. Green arrows = positive relationship between delay discounting log_10_(k) scores and effective connectivity, red arrows = negative relationship between delay discounting log_10_(k) scores and effective connectivity. Yellow shading = simple main effects in CD only, blue shading = simple main effects in HC only.

The PEB second-level GLM results are reported in Supplementary Table 3 and shown in Figure 2, and the analysis showed strong evidence for a positive relationship between delay discounting (log_10_(k) scores) and effective connectivity for the RECN to LECN pathway (Pp = 1.00) and for the DMN to DMN self-connection (Pp = 1.00). A PEB second-level GLM analysis showed strong evidence for a negative relationship between log_10_(k) scores and effective connectivity for the DMN to RECN pathway (Pp = 1.00) and for the SN to DMN pathway (Pp = 1.00).

A PEB second-level GLM analysis showed strong evidence for heterogeneous regression slopes between groups for log_10_(k) scores on the SN to RECN effective connectivity pathway (Pp = 1.00) and the RECN to SN effective connectivity pathway (Pp = 1.00). Therefore, we further examined these pathways by analyzing the simple main effects of log_10_(k) scores within CD only and by analyzing the simple main effects of log_10_(k) scores within HC only. The simple main effects analysis within CD showed strong evidence for a positive relationship between log_10_(k) scores and effective connectivity for the SN to RECN pathway (Pp = 1.00) and strong evidence for a negative relationship between log_10_(k) scores and effective connectivity for the RECN to SN pathway (Pp = 1.00). The simple main effects analysis within HC subjects showed strong evidence for a positive relationship between log_10_(k) scores and effective connectivity for the RECN to SN pathway (Pp = 1.00).

##### 3.3.2.4. DCM PEB results – UDS

The PEB second-level GLM results are reported in Supplementary Table 4 and the analysis showed strong evidence for positive main effects of cocaine-positive UDS different from zero on the effective connectivity for the LECN to SN pathway (Pp = 1.00), for the LECN to RECN pathway (Pp = 1.00), and the SN to SN self-connection pathway (Pp = 1.00). Results also showed strong evidence for negative main effects of cocaine-positive UDS different from zero on the effective connectivity for the SN to RECN pathway (Pp = 1.00), for the DMN to LECN pathway (Pp = 1.00), and for the DMN to DMN self-connection (Pp = 1.00).

PEB second-level GLM analysis showed strong evidence for positive main effects of cannabis-positive UDS different from zero on the effective connectivity for the LECN to RECN pathway (Pp = 1.00).

##### 3.3.2.5. DCM PEB multifactorial GLM results – Current tobacco use status

**FIGURE 3:**
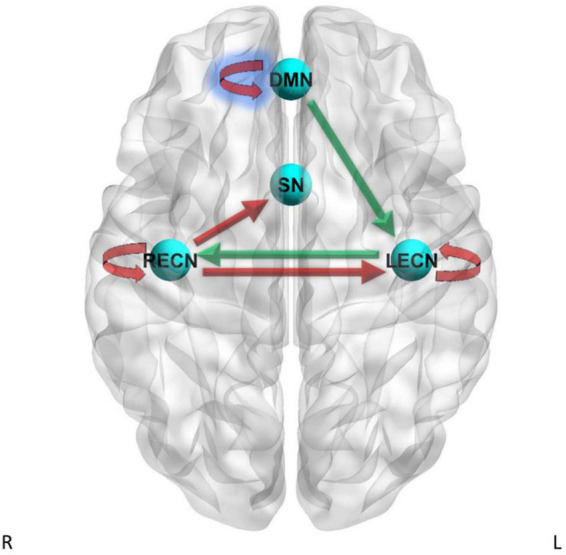
Dynamic causal modeling PEB results – current tobacco use status. Green arrows = current tobacco use > non-current tobacco use, red arrows = current tobacco use < non-current tobacco use. Blue shading = simple main effects in HC only.

**FIGURE 4:**
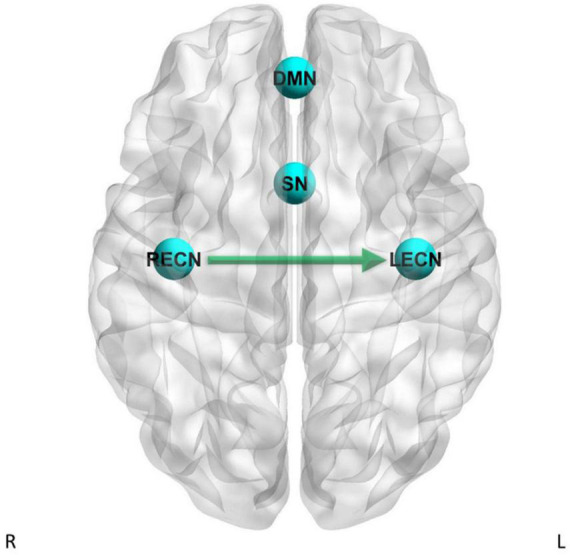
Dynamic causal modeling PEB results – group differences with current tobacco use status as factor in the GLM. Green arrow = CD has stronger effective connectivity than HC.

PEB Multifactorial GLM was conducted with current tobacco use status included in the model as a factor in addition to group and the interaction of group x current tobacco use status. The results are reported in Supplementary Tables 5, 6 and shown in Figures 3, 4. For the DMN to LECN pathway there was strong evidence (Pp = 1.00) for the effects of current tobacco use greater than non-current tobacco use on effective connectivity, and no strong evidence of a difference between CD and HC on effective connectivity for this pathway. For the LECN to RECN pathway, there was strong evidence (Pp = 1.00) for the effects of current tobacco use greater than non-current tobacco use on effective connectivity, and no strong evidence of a difference between CD and HC on effective connectivity for this pathway.

For the LECN to LECN self-connection, there was strong evidence (Pp = 1.00) for the effects of current tobacco use less than non-current tobacco use on effective connectivity, and no strong evidence of a difference between CD and HC on effective connectivity. For the RECN to SN pathway, there was strong evidence (Pp = 1.00) for the effects of current tobacco use less than non-current tobacco use on effective connectivity, and no strong evidence of a difference between CD and HC on effective connectivity. For the RECN to LECN pathway, there was strong evidence (Pp = 1.00) for the effects of current tobacco use less than non-current tobacco use on effective connectivity, and also strong evidence for CD to have greater effective connectivity than HC (Pp = 1.00; difference = 0.0846 Hz) for this pathway. For the RECN to RECN self-connection, there was strong evidence (Pp = 1.00) for the effects of current tobacco use less than non-current tobacco use on effective connectivity, and no strong evidence of a difference between CD and HC on effective connectivity.

The PEB second-level multi-factor GLM analysis showed strong evidence for an interaction between current tobacco use x group for the LECN to DMN effective connectivity pathway (Pp = 1.00) and for the DMN to DMN self-connection (Pp = 1.00). Therefore, we further examined these pathways by analyzing the simple main effects of current tobacco use within CD subjects only and by analyzing the simple main effects of current tobacco use within HC subjects only. The simple main effects analysis within HC subjects showed strong evidence for negative effects of current tobacco use different from zero on the effective connectivity for the DMN to DMN self-connection (Pp = 1.00). The simple main effects analysis within the CD group did not show strong evidence for the effects of current tobacco use status for the DMN to DMN self-connection. For the LECN to DMN pathway, there was no strong evidence for the effects of current tobacco use status within the CD group and also no strong evidence for effects of current tobacco use status within the HC group.

## 4. Discussion

### 4.1. Delay discounting and effective connectivity

The PEB analysis showed strong evidence for a positive relationship between delay discounting and effective connectivity for the RECN to LECN pathway and a negative relationship between delay discounting and effective connectivity for the SN to DMN and for the RECN to DMN pathways. CD and HC showed a contrasting relationship between delay discounting and RECN to SN effective connectivity, with CD showing strong evidence for a positive relationship and HC showing strong evidence for a negative relationship. CD also showed strong evidence for a positive relationship between delay discounting and SN to RECN effective connectivity, while HC did not. CD in our study scored significantly higher in delay discounting compared to HC, but our results did not support an association between RECN functional connectivity and delay discounting. Delay discounting has previously been associated with within-network resting state fMRI RECN functional connectivity (23), but to our knowledge has not been studied in association with between-network resting state fMRI RECN functional or effective connectivity.

### 4.2. Tobacco use and effective connectivity

Tobacco use also showed strong evidence for a positive relationship with RECN to LECN effective connectivity. Additionally, when current tobacco use status was controlled statistically as a factor in the model there was strong evidence for group differences in RECN to LECN effective connectivity, with CD having stronger effective connectivity than HC. Tobacco use is common in CD and other substance use disorders (52), making it difficult to separate the effects of tobacco use from the effects of the substance use disorder. Reese et al. (15) found stronger functional connectivity between the SN and RECN in a group of cocaine use disorder subjects with reported “moderate” tobacco use relative to non-cocaine using control subjects with no reported tobacco use. However, Reese et al. (15) did not separately analyze the effects of tobacco use on functional connectivity.

### 4.3. Cocaine- and cannabis-positive UDS and effective connectivity

Our results also showed strong evidence for a positive association between LECN to RECN effective connectivity and both UDS positive for cocaine and UDS positive for cannabis. Consistent with these findings, McCarthy et al. (53) found a positive relationship between the amount of cocaine metabolites measured by UDS, and a negative relationship between time since last cocaine use and functional connectivity between the LECN and RECN.

### 4.4. Summary

Taken together, these results suggest that effective connectivity of the RECN may relate to CD, recent cocaine or cannabis use, tobacco use, and delay discounting. The RECN has previously been linked to delay discounting, as well as to working memory and action-inhibition (22–24). Higher delay discounting has been shown in addictions, including in cocaine users (54–56), and CD had higher delay discounting log_10_(k) scores than HC in the present study. Delay discounting scores are proposed to relate to increased substance use by increased preference for the immediate reward associated with substance use, along with reduced consideration of the long-term negative consequences of substance use (28, 57). The RECN has been associated with executive functions (22–24) and, while speculative, may be “overactive” and have greater connectivity with other networks during the resting state in CD, tobacco users, and high impulsivity individuals. While it is difficult to isolate the specific effects of cocaine use, tobacco use, and delay discounting on RECN functional and effective connectivity due to their co-occurrence, our results suggest RECN functional and effective connectivity should be further studied in association with these factors in CD. Future studies should investigate the relationship between RECN functional and effective connectivity and treatment response in CD.

### 4.5. Negative findings

Our results failed to replicate the Goulden et al. (20) study which showed that the SN drove the switching between the DMN and ECN in HC. Our study employed an ICA approach which estimated LECN and RECN components, whereas the Goulden et al. (20) study estimated a single combined ECN. It is also possible that a particular node within the SN, such as the dorsal anterior cingulate cortex, is driving the switching between the ECN and DMN and that our whole network analysis was not sensitive to those region-specific effects. The second-level PEB analysis did not show strong evidence for any group differences in effective connectivity among the networks examined. However, there was strong evidence for a relationship between multiple effective connectivity pathways and current tobacco use, which was unbalanced between groups. Furthermore, after controlling statistically for current tobacco use by including current tobacco use as a factor in a multifactorial GLM, there was strong evidence for CD having stronger effective connectivity than HC in the RECN to LECN effective connectivity pathway. However, given that there were no group differences in effective connectivity without including current tobacco use status as a factor in the model and that current tobacco use status was imbalanced between groups such that most of the CD subjects were current tobacco users, interpretation of the group difference in the RECN to LECN effective connectivity pathway when current tobacco use status was included as a factor in the model should be interpreted with caution.

Our results did not show significant differences in LECN functional connectivity between groups, contrary to previous research in substance use disorder subjects (14–17). It is possible that our sample size was too small to detect a difference. Our results also did not show significant differences in RECN, DMN, or SN functional connectivity between groups. DMN functional connectivity differences in substance use disorder subjects have been shown previously (12). It is possible that different sub-systems and sub-regions of the DMN may be differentially affected by chronic drug use (12). Our results also did not show significant differences in between-network functional connectivity between groups for any of the 6 possible network pairings. It is possible that between-network functional and effective connectivities among the SN, DMN, LECN, and RECN are primarily through connectivities between specific subregions within each network, and thus a whole-network analysis may not be sensitive to these effects.

While our results showed strong evidence for a relationship between multiple effective connectivity pathways and the behavioral variables log_10_(k) scores and tobacco use status, our results did not show any significant relationships between the behavioral variables log_10_(k) and tobacco use status and between-network functional connectivity for any of the possible 6 network pairings. Recent work has shown that the reliability of effective connectivity may be greater than that of functional connectivity (58–60). Additionally, Bayesian Model Reduction (BMR) and the Empirical Bayes approach, both employed in this study, may further improve reliability of effective connectivity parameter estimates (58, 61, 62). Additionally, the PEB approach used in this analysis entails taking the averaged connectivity parameters at the group level from the first level up to the second level as updated priors, which may improve the sensitivity to detect an effect (27). It is therefore possible that the PEB effective connectivity analysis was able to detect an effect in our relatively small sample but that the sample was too small for the recommended classical frequentist approach applied in the functional connectivity analysis to detect an effect. Additionally, there is not a one-to-one linear relationship between effective connectivity and functional connectivity (4). Dynamic Causal Modeling-estimated effective connectivity is directional while functional connectivity is not directional. It is possible for there to be strong evidence for a positive relationship between the behavioral variable log_10_(k) and effective connectivity for a given pathway (i.e., effective connectivity from network “A” to network “B”), but a neutral or negative relationship between log_10_(k) scores and effective connectivity for the reciprocal pathway (i.e., effective connectivity from network “B” to network “A”), and these opposing effects could reduce the chance of detecting a statistically significant relationship between log_10_(k) scores and functional connectivity in a directionless functional connectivity analysis. While the results of the non-linear Stochastic Dynamic Causal Modeling analysis were negative, the non-linear Stochastic Dynamic Causal Modeling analysis only assessed whether there was evidence for non-linear modulatory effects of one network on the effective connectivities between the other networks, which is fundamentally different from the Spectral Dynamic Causal Modeling analysis which only assessed the effective connectivities between networks. The absence of non-linear modulatory effects does not preclude the presence of strong evidence for effective connectivity between networks.

### 4.6. Limitations

A limitation of our study was the small sample size, and thus replication of our results with a larger number of subjects is needed. Additionally, while the two groups did not significantly differ in sex composition, our sample contained more male subjects than female subjects. Within-SN functional connectivity showed a significant relationship with mFD. However, mFD did not significantly differ between groups and all subjects in both groups met the Parkes et al. (39) stringent motion criteria for inclusion in the analysis. Additionally, correction for the effects of head motion on the fMRI signal was conducted using ICA-AROMA (42) and the CompCor method (43). Furthermore, group ICA components with possible motion-related noise were regressed out by the FSL dual regression procedure ((37), pp. 64–65). We did not find significant group differences in SN functional connectivity, and it is possible that head motion could have influenced those negative findings. The results involving effective connectivity pathways that include the SN should be interpreted with caution. These included multiple pathways involving the SN which showed relationships between effective connectivity and delay discounting, cocaine-positive UDS, and current tobacco use status. mFD was not statistically significantly related to functional connectivity within the other 3 networks or 6 possible network pairs examined. Nevertheless, given the significant within-SN functional connectivity-mFD relationship, our effective connectivity results involving pathways to and from the SN should be interpreted with caution. In addition, the absence of strong evidence for the SN modulating the switching between the DMN and ECN should also be interpreted with caution. Another limitation is that the two groups significantly differed in delay discounting log_10_(k) scores and the number of subjects classified as current tobacco users. Given that the second-level PEB analysis showed strong evidence for a relationship between delay discounting log_10_(k) scores and current tobacco use status and multiple effective connectivity pathways, the imbalance in delay discounting log_10_(k) scores and current tobacco use status between groups may have confounded our results. Furthermore, while our results showed strong evidence for an association between delay discounting log_10_(k) scores and multiple effective connectivity pathways, our study did not include task-based fMRI data and therefore did not directly evaluate the effects of delay discounting on fMRI brain activations.

## Data availability statement

The raw data supporting the conclusions of this article will be made available by the authors, without undue reservation.

## Ethics statement

The studies involving human participants were reviewed and approved by the Virginia Commonwealth University Institutional Review Board. The patients/participants provided their written informed consent to participate in this study.

## Author contributions

FM and JS designed the studies from which subjects were sampled. JS, FM, LM, EZ, and KW designed the analyses for this study. ML reviewed the anatomical scans for incidental pathology. KW wrote the original manuscript draft. All authors reviewed, contributed to edits, and approved the final manuscript.

## Footnotes

1
www.fmrib.ox.ac.uk/fsl/

2
https://fsl.fmrib.ox.ac.uk/fsl/fslwiki/FSLNets

3
www.nitrc.org/projects/conn

4
https://fsl.fmrib.ox.ac.uk/fsl/fslwiki/MELODIC

5
https://fsl.fmrib.ox.ac.uk/fsl/fslwiki/PALM

6
https://fsl.fmrib.ox.ac.uk/fsl/fslwiki/Atlases
