# Supplementary material for: Executive control network resting state fMRI functional and effective connectivity and delay discounting in cocaine dependent subjects compared to healthy controls
Source: Front Psychiatry. 2023 Feb 23;14:1117817. doi: 10.3389/fpsyt.2023.1117817 (PMC9997846; doi:10.3389/fpsyt.2023.1117817)
Supplement: Supplementary file 1 [file Data_Sheet_1.docx]

## Supplementary Information

**SI-Table 1.** Dynamic Causal Modeling Stochastic Non-linear Bayesian Model Selection Results. PXP=protected exceedance probability, BOR=Bayesian Omnibus Risk, L=left, and R=right.

|  | HC subjects (n=22) | | CD subjects (n=22) | |
| --- | --- | --- | --- | --- |
| Putative nonlinear modulator | PXP | BOR | PXP | BOR |
| SN | 0.2000 | 0.98 | 0.1989 | 0.97 |
| DMN | 0.1993 | 0.98 | 0.1993 | 0.97 |
| LECN | 0.2002 | 0.98 | 0.2011 | 0.97 |
| RECN | 0.2004 | 0.98 | 0.2005 | 0.97 |
| LECN & RECN | 0.2002 | 0.98 | 0.2001 | 0.97 |

**SI-Table 2.** The results of the Dynamic Causal Modeling PEB analysis for comparison between CD and HC. EC=effective connectivity, PP=posterior probability, L=left, and R=right.

|  | Main effects across all CD and HC subjects combined (n=44) | | Group differences: CD (n=22) – HC (n=22) | |
| --- | --- | --- | --- | --- |
| Connectivity | EC (Hz) | PP | EC (Hz) | PP |
| SN→SN | -0.4768 | 1 | 0.0005 | 0 |
| SN→DMN | -0.0729 | 0.9932 | 0.0035 | 0 |
| SN→LECN | 0.0016 | 0 | 0.0049 | 0 |
| SN→RECN | 0.0031 | 0 | 0.0017 | 0 |
| DMN→SN | 0.0268 | 0.5830 | -0.0014 | 0 |
| DMN→DMN | -0.3678 | 1 | -0.0020 | 0 |
| DMN→LECN | -0.0028 | 0 | -0.0008 | 0 |
| DMN→RECN | -0.1540 | 1 | 0.0024 | 0 |
| LECN→SN | -0.0022 | 0 | 0 | 0 |
| LECN→DMN | -0.0025 | 0 | -0.0007 | 0 |
| LECN→LECN | -0.0524 | 0.6186 | 0.0010 | 0. |
| LECN→RECN | -0.1106 | 0.9766 | 0.0004 | 0 |
| RECN→SN | -0.0451 | 0.7726 | -0.0029 | 0 |
| RECN→DMN | 0.0037 | 0 | 0.0030 | 0 |
| RECN→LECN | 0.0703 | 0.9136 | 0.0036 | 0 |
| RECN→RECN | -0.3747 | 1 | -0.0011 | 0 |

**SI-Table 3.** The results of the Dynamic Causal Modeling PEB delay discounting regression analysis. EC=effective connectivity, PP=posterior probability, L=left, and R=right. Two subjects in each group did not have delay discounting task data recorded.

|  | Delay discounting regression across all CD and HC subjects combined (n=40) | | Delay discounting regression: CD only (n=20) | | Delay discounting regression: HC only (n=20) | |
| --- | --- | --- | --- | --- | --- | --- |
| Connectivity | EC (Hz) | PP | EC (Hz) | PP | EC (Hz) | PP |
| SN→SN | -0.0002 | 0 | -0.0008 | 0 | -0.0004 | 0 |
| SN→DMN | -0.0586 | 1 | -0.076 | 1 | -0.0716 | 1 |
| SN→LECN | 0.0036 | 0 | 0.0537 | 1 | 0.0005 | 0 |
| SN→RECN | -0.1165 | 1 | -0.1595 | 1 | -0.0522 | 0.831437834 |
| DMN→SN | 0.0006 | 0 | 0.0033 | 0 | -0.0006 | 0 |
| DMN→DMN | 0.1186 | 1 | 0.0501 | 0.669868156 | 0.1882 | 1 |
| DMN→LECN | -0.0439 | 0.848983791 | -0.0593 | 1 | -0.0539 | 1 |
| DMN→RECN | -0.0786 | 1 | -0.0829 | 1 | -0.0775 | 1 |
| LECN→SN | -0.0014 | 0 | 0.0017 | 0 | -0.0302 | 0.544560614 |
| LECN→DMN | 0.0021 | 0 | 0.0002 | 0 | 0.0741 | 0.895531554 |
| LECN→LECN | 0.0013 | 0 | -0.001 | 0 | 0.0985 | 0.875219845 |
| LECN→RECN | 0.0284 | 0.550269472 | 0.0997 | 1 | 0.0007 | 0 |
| RECN→SN | 0.058 | 1 | 0.0007 | 0 | 0.1119 | 1 |
| RECN→DMN | 0.0351 | 0.759451247 | 0.0031 | 0 | 0.0331 | 0.695749822 |
| RECN→LECN | 0.0841 | 1 | 0.0311 | 0.594178858 | 0.0949 | 1 |
| RECN→RECN | -0.0011 | 0 | -0.0879 | 0.90358629 | -0.0001 | 0 |

**SI-Table 4.** The results of the Dynamic Causal Modeling PEB Cocaine-positive and Cannabis-positive UDS analysis. EC=effective connectivity, PP=posterior probability, L=left, and R=right.

|  | Cocaine-positive UDS analysis in CD subjects (n=22) | | Cannabis-positive UDS analysis in CD subjects (n=22) | |
| --- | --- | --- | --- | --- |
| Connectivity | EC (Hz) | PP | EC (Hz) | PP |
| SN→SN | 0.1917 | 1 | -0.1253 | 0.68352765 |
| SN→DMN | -0.0074 | 0 | 0.0022 | 0 |
| SN→LECN | -0.0063 | 0 | 0.007 | 0 |
| SN→RECN | -0.1419 | 1 | -0.0012 | 0 |
| DMN→SN | -0.0043 | 0 | 0.002 | 0 |
| DMN→DMN | -0.1741 | 1 | 0.0031 | 0 |
| DMN→LECN | -0.1102 | 1 | 0.0082 | 0 |
| DMN→RECN | -0.0733 | 0.62470895 | 0.0022 | 0 |
| LECN→SN | 0.1317 | 1 | -0.0055 | 0 |
| LECN→DMN | 0.0067 | 0 | 0.0021 | 0 |
| LECN→LECN | 0.001 | 0 | -0.1027 | 0.59794362 |
| LECN→RECN | 0.1864 | 1 | 0.2484 | 1 |
| RECN→SN | -0.0117 | 0 | -0.0101 | 0 |
| RECN→DMN | -0.0061 | 0 | 0.0751 | 0.695891934 |
| RECN→LECN | 0.0044 | 0 | -0.054 | 0.501284651 |
| RECN→RECN | 0.0011 | 0 | -0.0662 | 0.509704878 |

**SI-Table 5.** The results of the Dynamic Causal Modeling PEB current tobacco use multifactorial model, PP=posterior probability, L=left, and R=right. Two CD subjects did not have tobacco use status data recorded.

|  | Current tobacco use greater than non-current tobacco use in all subjects combined (n=42) | | Group differences: CD (n=20) – HC (n=22) | | Group x current tobacco use status interaction (n=42) | |
| --- | --- | --- | --- | --- | --- | --- |
| Connectivity | EC (Hz) | PP | EC (Hz) | PP | EC (Hz) | PP |
| SN→SN | 0.0024 | 0 | 0.0006 | 0 | -0.0028 | 0 |
| SN→DMN | 0.0488 | 0.530968982 | 0.0004 | 0 | -0.0068 | 0 |
| SN→LECN | 0.0093 | 0 | 0.005 | 0 | -0.0012 | 0 |
| SN→RECN | 0.0044 | 0 | 0.0015 | 0 | 0.0061 | 0 |
| DMN→SN | -0.0089 | 0 | -0.0004 | 0 | 0.0064 | 0 |
| DMN→DMN | -0.2331 | 1 | 0.0007 | 0 | 0.2437 | 1 |
| DMN→LECN | 0.1536 | 1 | -0.0444 | 0.746422803 | -0.0052 | 0 |
| DMN→RECN | 0.1042 | 0.831760601 | -0.0011 | 0 | -0.1075 | 0.793233810516053 |
| LECN→SN | -0.0011 | 0 | 0.0006 | 0 | 0.0016 | 0 |
| LECN→DMN | -0.0003 | 0 | -0.0003 | 0 | 0.1913 | 1 |
| LECN→LECN | -0.4043 | 1 | 0.1119 | 0.852408348 | 0.0008 | 0 |
| LECN→RECN | 0.3897 | 1 | -0.0766 | 0.82419698 | 0.0026 | 0 |
| RECN→SN | -0.1386 | 1 | 0.002 | 0 | -0.0007 | 0 |
| RECN→DMN | 0.0011 | 0 | 0.002 | 0 | 0.0029 | 0 |
| RECN→LECN | -0.1801 | 1 | 0.0846 | 1 | 0.0044 | 0 |
| RECN→RECN | -0.3197 | 1 | 0.0004 | 0 | 0.1109 | 0.598723371244673 |

**SI-Table 6.** The Simple Main Effects results of the Dynamic Causal Modeling PEB current tobacco use status for pathways showing interaction of group x tobacco use status in SI-Table 5, PP=posterior probability, L=left, and R=right. Two CD subjects did not have tobacco use status data recorded.

|  | Current tobacco use greater than non-current tobacco use in CD only (n=20) | | Current tobacco use greater than non-current tobacco use in HC only (n=22) | |
| --- | --- | --- | --- | --- |
| Connectivity | EC (Hz) | PP | EC (Hz) | PP |
| DMN→DMN | 0.0003 | 0 | -0.4294 | 1 |
| LECN→DMN | 0.238 | 0.919271814 | -0.0042 | 0 |
